# A Potential Role for Nivolumab in the Treatment of Fibrous Dysplasia-Related Pain

**DOI:** 10.1210/jcemcr/luae165

**Published:** 2024-09-18

**Authors:** Mohammad Jay, Cassandra Hawco, Kristin K Clemens, Stan Van Uum

**Affiliations:** Division of Endocrinology, Department of Medicine, University of Toronto, Toronto, ON M5S 1A1, Canada; Division of Endocrinology, Department of Medicine, Western University, London, ON N6A 5C1, Canada; Division of Endocrinology, Department of Medicine, Western University, London, ON N6A 5C1, Canada; Division of Endocrinology, Department of Medicine, Western University, London, ON N6A 5C1, Canada

**Keywords:** fibrous dysplasia, RANK ligand, nivolumab

## Abstract

Fibrous dysplasia (FD) is a chronic and progressive disorder of bone growth because of decreased osteoblast formation and osteoclast overactivity. Its main symptoms include pain, fracture, and irregular bone growth. Bisphosphonates are the mainstay of therapy for FD with a primary goal of pain relief. A 50-year-old woman presented to ophthalmology in March 2011 with intermittent proptosis, vertical diplopia, and orbital pain. A computed tomography scan of the head revealed a skull base lesion, which was confirmed to be fibrous dysplasia on bone biopsy. Because of significant headache, she was treated with IV pamidronate monthly starting November 2011, which led to pain reduction. Repeated attempts to decrease the frequency of pamidronate were unsuccessful because of breakthrough pain. Oral alendronate and risedronate did not control her symptoms. She remained on risedronate however because of its convenience. In August 2021, she was diagnosed with metastatic melanoma and started nivolumab. Her headache completely resolved for the first time in 10 years. Although nivolumab, a programmed death-1 blocker, has been used in the treatment of bone malignancy, it has not been previously studied in FD. By suppressing RANK ligand-related osteoclastogenesis, nivolumab decreases cancer-associated bone pain. Our case suggests a potential role for nivolumab in treating FD-associated pain.

## Introduction

Fibrous dysplasia (FD) is a progressive disorder of bone development, constituting 7% of benign bone tumors [1]. Common symptoms include pain, pathological fracture, and irregular bone growth. The ribs, pelvis, and craniofacial bones are most frequently affected. FD can also be associated with other endocrinopathies, including hyperthyroidism, hypophosphatemic rickets, acromegaly, hypercortisolism, and hyperprolactinemia [1].

The histological hallmark of FD is extensive fibro-osseous tissue growth, replacing the normal lamellar cancellous osseous tissue. This is due to a gain of function mutation of a G protein-coupled receptor, guanine nucleotide-binding protein alpha stimulating mutation, leading to increased production of cyclic adenosine monophosphate. This drives rapid proliferation of the osteoblast progenitor cells, preventing their full maturation. In parallel, the guanine nucleotide-binding protein alpha stimulating mutation leads to increased IL-6 production, which enhances osteoclastogenesis [1].

FD treatment is usually conservative, unless the lesions are deemed high risk for fracture, for which case surgery is considered. Medical therapy is used for those who are ineligible for surgery and/or to manage the FD-related pain [1]. Bisphosphonates are the mainstay of medical therapy [1]. The RANK ligand (RANK-L) inhibitor, denosumab, is considered second-line treatment in patients refractory to bisphosphonates [2]. Bisphosphonates and denosumab reduce bone pain through inhibiting osteoclast maturation and bone resorption [3]. Corticosteroids are used when visual loss is present [4].

Nivolumab, a human programmed death-1 (PD-1) blocking antibody, has been used in the treatment of bone-related malignancy [5]. Here, we report a case of a patient with FD and metastatic melanoma whose fibrous dysplasia pain improved following melanoma treatment with nivolumab.

## Case Presentation

In March 2011, a 51-year-old woman developed double vision, right temporal headache, as well as right eye pain and swelling. Her medical history included glaucoma and perimenopause. She was assessed by ophthalmology and further imaging was obtained for diagnosis.

## Diagnostic Assessment

**Figure 1.**
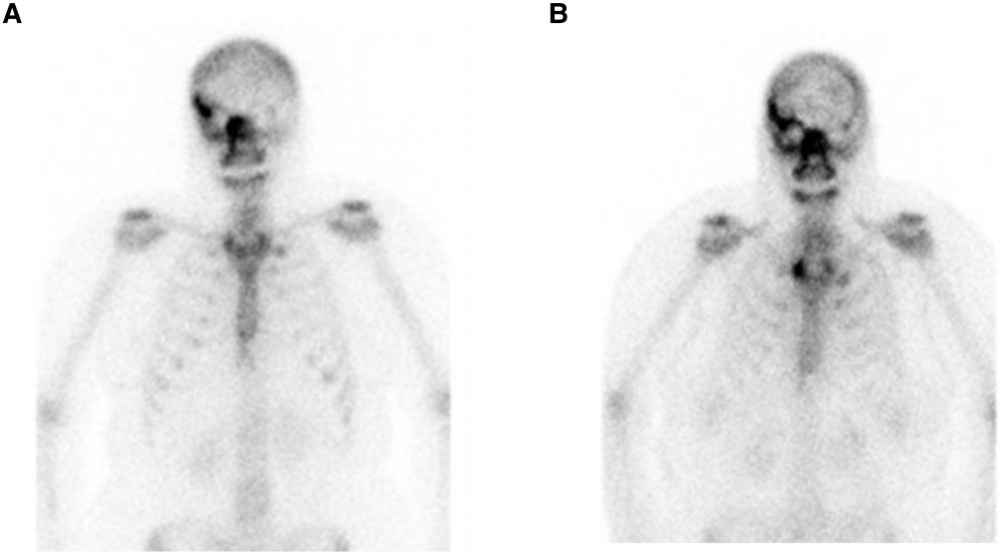
Bone scan images at diagnosis in 2011 (A) and most recently in 2022 (B). Prominent uptake of the right orbit and the right skull base consistent with fibrous dysplasia of the right facial bones. No evidence of fibrous dysplasia outside of the skull. The most recent images show stable right-sided facial fibrodysplasia.

A magnetic resonance imaging scan identified thickening of the right lateral rectus muscle, as well as thickening of the right sphenoid bone and extracoronal soft tissues. A computed tomography scan of the head showed reduced bone density of the sphenoid temporal bone and mild bony expansion of the posterior right orbit. Pathology from biopsy of the right pterygoid was indicative of fibrous dysplasia. The patient had a bone scan that did not identify any other sites of FD (Fig. 1A). Furthermore, dual-energy X-ray absorptiometry scan was normal. She was found to have mild vitamin D deficiency with a 25-hydroxy vitamin D level of 50 (reference range, 75-250 nmol/L) nmol/L (20 ng/mL), but other laboratory testing including alkaline phosphatase and calcium studies were within normal limits.

## Treatment

Because her pain was tolerable on initial presentation, treatment with bisphosphonate was not initiated for the first year following diagnosis. Due to increasing headache and proptosis, she began 90 mg pamidronate IV monthly in November 2011, which resulted in improvement in her headache. Attempts to decrease the frequency of pamidronate dosing were unsuccessful, as was transitioning to oral alendronate that was trialed for patient convenience. In July 2021, to reduce travelling required for monthly pamidronate injections, she was transitioned to 35 mg oral risedronate weekly. Two months earlier, in May 2021, she was diagnosed with melanoma with metastases to the lung and liver. Nivolumab was initiated in August 2021. She did not receive any other therapy with the nivolumab, specifically no glucocorticoids except for chronic use of budesonide/formoterol inhalations.

## Outcome and Follow-up

She has remained pain free since the fall of 2021 and continues to take risedronate 35 mg weekly. Her most recent bone scan in March 2022 demonstrated mildly methylene diphosphonate-avid FD, which is stable in size (Fig. 1B). As of November 2023, she completed 30 treatment doses of nivolumab, with stability of her melanoma.

## Discussion

Here, we report a unique case demonstrating improvement in FD-related pain following treatment with a PD-1 blocker, nivolumab. PD-1 blockers belong to a group of medications known as immune checkpoint inhibitors and are widely used for the treatment of malignancies, including metastatic melanoma. PD-1 regulates the immune system inflammatory response, with its increased expression tied to immune suppression. Cancer cells often enhance PD-1 expression, allowing them to evade the immune system by suppressing the immune response [6].

PD-1 receptors have been shown to be expressed by sensory neural cells in murine models with bone cancer pain. Treatment with PD-1 blockers led to a brief initial increase in pain sensitivity, followed by a subsequent long-term improvement in cancer bone pain [5]. We suggest that the mechanism through which nivolumab improves FD-related pain could also be related to RANK-L inhibition. RANK-L acts by activating osteoclasts, leading to bone erosion. PD-1 promotes osteoclastogenesis through RANK-L activation and PD-1 blockers have been shown to inhibit osteoclastogenesis [5]. Denosumab has been shown to reduce FD pain by acting through the same mechanism [2]. Previous animal studies in models with FD have demonstrated decreased growth of irregular bone tissues and increased bone mineralization following treatment with denosumab [2]. Whether PD-1 receptors are expressed in pain receptor neural cells in FD has not been studied; however, using the cancer bone models, we stipulate that PD-1 blockers inhibit FD pain through both inhibition of osteoclastogenesis and sensory neuron signalling.

Our patient's initial imaging demonstrated radiographic changes in the adjacent soft tissue and the ocular muscle. Nivolumab may have reduced swelling, inflammation, and associated pain. Soft tissue involvement is uncommon in FD, but previous reports of FD of sphenoid with soft tissue involvement demonstrated improvement in pain following excision of the edematous tissues and optic nerve decompression [7-9]. Immune checkpoint inhibitors such as PD-1 inhibitors have been shown to reduce inflammation in the nervous and pulmonary systems of animal models [10, 11]. Similarly, a systematic review demonstrated immune checkpoint inhibitors were effective in the treatment of soft tissue sarcoma [12]. Moreover, sarcomas of an “inflamed phenotype” were more likely to respond to immune checkpoint inhibitors [13]. Of note, bisphosphonates do not have a significant anti-inflammatory effect and have not been shown to reduce soft tissue edema. In fact, there are reports of bisphosphonate-induced orbital inflammation [14].

It is interesting that posttreatment, the FD radiographic features remained stable. A phase III study assessing 20 patients with FD demonstrated improvement in the X-rays of all affected sites, pain, and bone turnover markers following treatment with IV pamidronate [15]. Conversely, a prospective study of 7 patients with FD receiving IV pamidronate did not show change in X-rays despite improvement in bone pain and bone turnover markers [16]. Similar discrepant results in terms of radiographic improvement have been observed in studies of patients with FD who were treated with alendronate [17, 18]. Therefore, our findings are not unexpected.

Our case suggests a potential beneficial effect of nivolumab in treating FD pain. Given the less desirable side effect profile of PD-1 blockers compared to bisphosphonates and denosumab, PD-1 blockers should not be considered as a potential first-line therapy for FD pain, however. The main side effects of immune checkpoint inhibitors include inflammatory responses in 1 or multiple organs. The prevalence of these side effects among the oncology population is approximately 10% and their frequencies depend on the length of exposure, host factors (eg, comorbidities), and the type of PD-1 blocker used [19]. In contrast, the most common side effect of bisphosphonates are gastrointestinal symptoms occurring in 14% to 20% of patients [20]. Less frequent, but more serious side effects of bisphosphonates include atypical femoral fracture and osteonecrosis of the jaw.

Two limitations were present in terms of evaluation and management of our patient: (1) turnover markers, which could have further clarified the effectiveness of nivolumab plus a bisphosphonate compared to a bisphosphonate alone, were not performed; and (2) nivolumab was started around the same time as switching pamidronate to risedronate. We concluded the improvement in bone pain was related to initiating nivolumab given that previous oral bisphosphonates were less effective. However, additional beneficial effects from risedronate compared to other bisphosphates cannot be definitively ruled out. Future preclinical and phase 0 trials could investigate the role of PD-1 blockers in FD. These studies may help to understand the molecular mechanism of PD-1 blockers in treating FD pain, which can be elucidated and harnessed to develop targeted therapy for pain related to FD and bone diseases with a similar pathophysiology.

## Learning Points

- Identify the pathophysiology, clinical presentation, diagnosis, and first-line option for management of fibrous dysplasia.
- Recognize the role of RANK ligand-related osteoclastogenesis inhibition as the mechanism involved in reducing bone pain in cancer-related bone pain and its potential role in reducing fibrous dysplasia-related bone pain.
- In broad terms, contrast the side effect profiles of programmed death-1 inhibitors and antiresorptive medications.

## Data Availability

Data sharing is not applicable to this article as no datasets were generated or analyzed during the current study.

## Abbreviations

FD: fibrous dysplasia
PD-1: programmed death-1
RANK-L: RANK ligand
